# Objective evaluation of oxaliplatin-induced vascular pain secondary to peripheral vein administration

**DOI:** 10.1186/s40064-016-3579-1

**Published:** 2016-10-26

**Authors:** Yoichiro Yoshida, Ai Mogi, Naoya Aisu, Teppei Yamada, Taisuke Matsuoka, Daibo Kojima, Toshiyuki Mera, Tomoko Koganemaru, Fumiaki Kiyomi, Keita Noda, Yasushi Takamatsu, Kazuo Tamura, Yuichi Yamashita, Suguru Hasegawa

**Affiliations:** 1 Department of Gastroenterological Surgery, Fukuoka University Faculty of Medicine, 7-45-1 Nanakuma, Jonan-ku, Fukuoka, 814-0180 Japan; 2 Division of Oncology, Hematology and Infectious Diseases, Department of Internal Medicine, Fukuoka University Faculty of Medicine, Fukuoka, Japan; 3 Academia, Industry and Government Collaborative Research Institute of Translational Medicine for Life Innovation, Fukuoka University, Fukuoka, Japan; 4 Clinical Research Assist Center, Fukuoka University Hospital, Fukuoka, Japan

**Keywords:** Chemotherapy, Colorectal cancer, Oxaliplatin, Vascular pain, PainVision

## Abstract

**Background:**

During oxaliplatin chemotherapy administration via a peripheral vein, vascular pain requires changing of the intravenous infusion route on occasion. Vascular pain induced by anticancer drugs reduces the rate of patient continuation and completion of chemotherapy. Pain is presently appraised using subjective methods, such as the visual analog scale (VAS). However, because pain evaluation can vary depending on the physical state and mood of the patient at the time of assessment, it is desirable to evaluate pain objectively. PainVision PS-2100 (PV) is a medical device that was designed to objectively and quantitatively assess patient nociception and perception.

**Methods:**

The present study examined the correlation of subjective and objective assessment of oxaliplatin-induced vascular pain using VAS and PV, respectively.

**Results:**

Vascular pain was assessed using both PV and VAS a total of 173 times for 58 colorectal cancer patients. Partial correlation analysis was performed to evaluate the relationship between PV and VAS. The mean PV and VAS scores were 44.5 (range: 0–596) and 24.8 (range: 0–100), respectively. The partial correlation coefficient was 0.408 (p < 0.0001).

**Conclusions:**

A strong correlation was not observed between the results, and a weak correlation was observed between VAS and PV scores. Objective evaluation of oxaliplatin-induced vascular pain will be required to help patients overcome vascular pain.

## Background

FOLFIRI (irinotecan with folinic acid and fluorouracil) and FOLFOX (oxaliplatin with folinic acid and fluorouracil) therapies for colorectal cancer (CRC) require the use of central venous (CV) ports. Recently developed therapies combining capecitabine plus oxaliplatin (XELOX) allow for chemotherapy via a peripheral vein without CV port (Yoshida et al. 2015b). However, vascular pain (VP) occasionally necessitates the movement of oxaliplatin drip infusions to a peripheral vein during XELOX therapy. VP following intravenous infusion of anticancer drugs can be caused by osmotic pressure and solution pH ultimately having a negative impact on the continuation of chemotherapy (Kuwahara et al. 1998; Yoshida et al. 2012).

Pain is complicated human experience, and tissue damage is not the only determinant of the extent of pain, which is a subjective, personal, and multidimensional experience, and not merely a simple sensation (Turk and Melzack 1992). Pain processes do not begin with the stimulation of receptors. Rather, injury or disease produces neural signals that enter an active nervous system that is the substrate of past experience, culture, and a host of other environmental and personal factors (Melzack and Katz 2013). Pain is presently estimated using subjective methods, such as the visual analog scale (VAS) (McCormack et al. 1988; DeLoach et al. 1998; Fishbain et al. 2016). VAS is a subjective self-assessment method for grading pain relative to the most intense pain that the patient has ever experienced (Babul et al. 1993; Huguet et al. 2010). Because of the usability, VAS has become a common tool for the quantification of pain intensity and pain relief. This method has proven to be a valid and reliable means to assess depression, pain, mood, and anxiety (McCormack et al. 1988; Lener et al. 2016). VAS consists of a line having a predetermined length and direction, with bilateral limits that indicate absence or minimum amount of the cited phenomenon on one end, and maximum or full amount of the phenomenon on the other. Verbal rating scale (VRS), has a variable number of gradually ascending verbal descriptors; the other approach, called a numeric rating score (NRS), also has a variable number of categories, most commonly 5 or 10 (Lara-Munoz et al. 2004). NRS and VRS are insufficient to evaluate VP because the methods cannot differentiate small changes in pain (Hjermstad et al. 2011; Mujezinovic and Alfirevic 2011). NRS and VRS thus allow only a less-subtle distinction of pain levels compared with PainVision™ PS-2100 system (PV; Nipro Co., Osaka, Japan)/VAS, where there are a theoretically unlimited number of possible answers. Therefore, we chose VAS to assess VP. However, researchers have reported that 7–20% of patients are unable to complete VAS (Kremer et al. 1981; Revill et al. 1976; Gregory 2012). Evaluation of pain can vary depending on the physical state and mood of the patient at the time of evaluation; thus, it is desirable to objectively evaluate pain. Pain evaluation by VAS is related with a margin of error of about ±20 mm (DeLoach et al. 1998). Therefore, a method to objectively assess pain is also required when evaluating drugs designed to improve VP.

The PV was developed in clinical practice for quantitative analysis of pain perception (Matsumura et al. 2012; Lee et al. 2014; Ohtori et al. 2014; Hiraki et al. 2014; Kim et al. 2014; Fukada et al. 2011; Yoshida et al. 2015c). PV is an analytical device that was designed to objectively and quantitatively assess patient sense nociception and perception. The PV apparatus can stimulate Aβ and Aδ fibers, and the degree of pain is calculated from two variables, the current perception threshold and current producing comparable pain as measured by this instrument. Its advantages include the ability to evaluate pain relatively rapidly and without causing additional pain to the patient. Although we reported that VAS and PV are useful to evaluate oxaliplatin-induced peripheral neuropathy with the aim of aiding treatment (Yoshida et al. 2015c), there have been no reports concerning the use of these measures for the assessment of VP due to oxaliplatin. Therefore, the purpose of this study was to identify potential correlations between the results of subjective and objective assessment methods to evaluate oxaliplatin-induced VP as measured using PV and VAS, respectively.

## Methods

### Patients

The institutional review board at Fukuoka University approved the protocol (Approval no: 13-4-07).

Patients with histologically confirmed metastatic CRC who had not received chemotherapy or who had completed adjuvant chemotherapy during the last 6 months were enrolled in this study. Patients with a poor mental health that prevented understanding of the concepts of PV and VAS were excluded. Patients were also excluded if they had experienced any musculoskeletal pain or peripheral sensory neuropathy prior to chemotherapy that may have disrupted the quantitative measurement of pain. Written informed consent was obtained from all patients.

### Chemotherapy

A total of 58 patients with metastatic CRC who underwent XELOX plus bevacizumab therapy (7.5 mg/kg bevacizumab and 130 mg/m^2^ oxaliplatin on day 1 plus 2000 mg/m^2^ capecitabine on days 1–14, repeated every 3 weeks) or XELOX therapy (130 mg/m^2^ oxaliplatin on day 1 plus 2000 mg/m^2^ capecitabine on days 1–14, repeated every 3 weeks) (Yoshida et al. 2015a, b) at Fukuoka University Hospital between April and August 2014 were included in this study.

### Data collection

VAS is a commonly used method to evaluate variations in pain intensity. Subjects were instructed to indicate the strength of pain at the time of assessment by marking on a 100 mm horizontal line anchored with “0 (no pain)” on the left edge and “100 (worst pain imaginable)” on the right edge (Yoshida et al. 2015c).

**Fig. 1:**
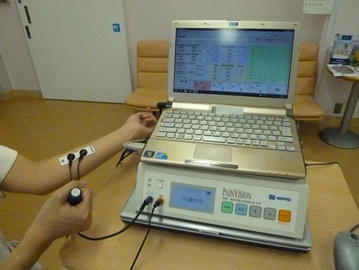
PV method of pain evaluation. Bipolar electrodes are attached to patients on the ulnar side of the forearm. Patients grasp a switch using their contralateral hand

For PV measurement, an electrode was attached to the medial side of the upper arm (Fig. 1) and an electric current of increasing amplitude is applied (50 Hz; 0–150 µA rms; pulse width: 0.3 ms). The patient was ordered to push a switch when this stimulation was perceived for the first time. The current at the point was defined as the “minimum perceived current” value. As the stimulation current was increased, the patient was ordered to push the button when the intensity of the stimulation current was equivalent to that of VP. The current at this point was defined as the “pain equivalent current” value. Using the values obtained, “pain intensity” was calculated using the aforementioned formula. When there was not a pain, the value was 0. The intensity and score then increased according to the degree of pain, with no upper limit. Each measurement was easy and can be performed within 2 min. The two pain assessments with VAS and PV were performed at the same time on the same set of patients (paired sampling), and the assessments were repeated within the same time interval (repeated measurements). VP assessment was performed by same nurse who received training.

### Data analysis

Data were analyzed using SAS software ver. 9.3 (SAS Institute, Cary, NC, USA). To evaluate the reliability of the device in terms of internal consistency, we assessed the quantitative pain degree score twice. Data are reported as the mean ± standard deviation, median (25–75% interquartile range), or number (percentage) of participants. Partial correlation analysis was carried out to estimate the relationship between VAS and PV after adjusting for subject and sex. In other words, the correlation coefficient between VAS and PV was calculated using residual values of the mixed-effects model including sex as a fixed effect and subject as a random effect. A probability (*p*) value of <0.05 was considered statistically significant.

## Results

A total of 64 patients received chemotherapy for metastatic CRC between April and August 2014. Six patients were excluded from the analysis according to the inclusion criteria (5 patients with ECOG PS 2 and one because of inadequate hematological, liver, and renal function). The final cohort included 18 women and 40 men (median age, 65 years; range 43–80 years). The characteristics of the study patients are shown in Table 1. Among the patients evaluated, 81.0% had ECOG PS 0, with the liver being the most common site of metastasis. The cumulative median dose of oxaliplatin was 1751 mg/body (range: 345–5903), and the number of cycles was 10. Grade 3–4 hematological toxicity and grade 3–4 non-hematological toxicity were recorded for 12.1 and 17.2% of patients, respectively.

**Table 1:** Baseline characteristics of patients who received chemotherapy

	N (%)	Mean	SD	Range
Mean age, years		65.2	8.6	43–80
*Gender*				
Male	40 (69.0)			
Female	18 (31.0)			
*Chemotherapy*				
XELOX	18 (31.0)			
XELOX + BV	40 (69.0)			
*ECOG PS*				
0	47 (81.0)			
1	11 (19.0)			
2	0 (0)			
3	0 (0)			
*Primary cancer*				
Colon	31 (53.4)			
Rectum	27 (46.6)			
*Adverse events (Grade 3*–*4)*				
Hematological	7 (12.1)			
Non-hematological	10 (17.2)			
Oxaliplatin (mg/body)		1751	136.6	345–5903

**Fig. 2:**
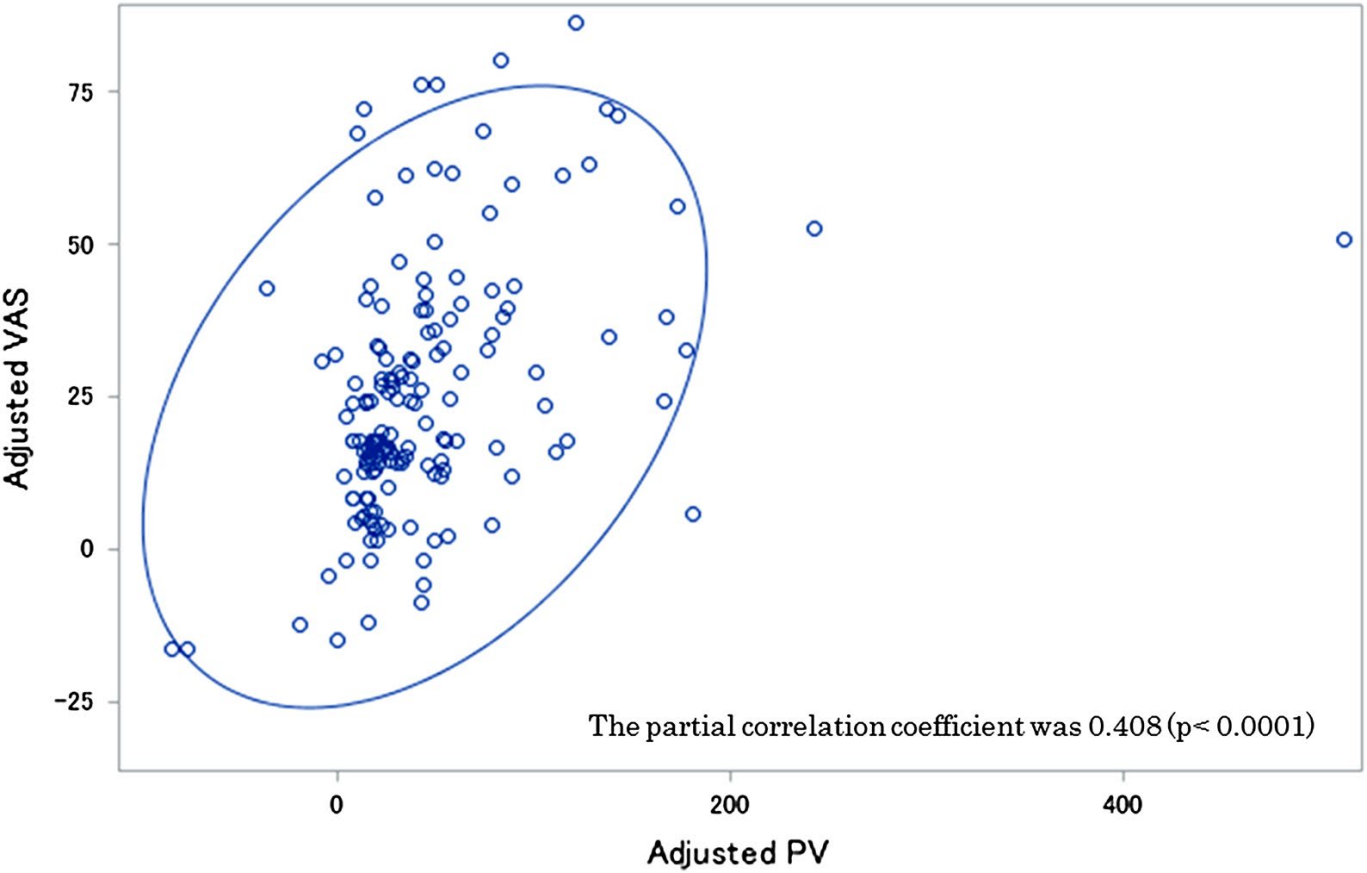
Correlation coefficient between VAS and PV scores adjusted for subject and sex

VP was assessed using both PV and VAS a total of 173 chemotherapy cycles. The mean VAS and PV scores for VP were 24.8 (range: 0–100) and 44.5 (range: 0–596), respectively. The partial correlation coefficients with no adjustment, after adjusting for subject and sex, and after exclusion of outliers (one patient) were 0.49 (*p* < 0.0001), 0.41 (*p* < 0.0001), and 0.45 (*p* < 0.0001), respectively (Table 2). A corresponding scatter plot and 95% prediction ellipse are shown in Fig. 2.

**Table 2:** Partial correlation coefficient analysis between VAS and PV

	Correlation coefficient	P value
No adjustment	0.490	<0.0001
Partial^a^	0.408	<0.0001
Exclusion of outlier^b^	0.450	<0.0001

^a^Partial correlation coefficient adjusted by subject and sex

^b^Partial correlation coefficient excluding outlier, PV = 596 (sensitivity analysis)

## Discussion

The correlation between VAS and PV to evaluate vascular pain related to oxaliplatin infusion is weak. However, it may be a matter of course. The pain is characterized by various dimensions including physical, social, and psychological; which together constitute the total pain. Total pain is not simply the end product of a linear sensory transmission system. VAS expresses the total pain, and PV expresses a physical pain. Therefore, it may be natural that there is no strong correlation between VAS and PV. It is thought that we should evaluate the physical pain using PV to improve the vascular pain.

We previously reported port-free chemotherapy administration via the median cubital vein for CRC and showed that the resulting low complication rates helped to ensure completion of chemotherapy without delay for most patients (Yoshida et al. 2012, b). However, VP occasionally necessitates changing an oxaliplatin drip infusion to a peripheral vein during XELOX therapy. Some investigators have reported that the addition of dexamethasone (DEX) to an oxaliplatin drip infusion can help control vascular pain (Matsuyama et al. 2011). We evaluated the effectiveness of DEX for controlling VP caused by oxaliplatin administration via a peripheral vein during XELOX therapy (Yoshida et al. 2012). However, VP did not completely disappeared. Therefore, we aimed to establish a new evaluation method of VP.

VAS tends to focus only on pain intensity, with an increased risk for over-simplification of the experience (Bonica et al. 1990). Furthermore, actual assessments are relative only to the individual being assessed. Same stimulation applied to different individuals can give remarkably different scores. Pain is a subjective, personal, and multidimensional experience rather than just a simple sensation (Turk and Melzack 1992; Burton et al. 2014). Therefore, pain evaluation is complicated process, and it is imperative that it takes into account the many factors that affect the experience of pain. The disadvantages of VAS are that patients oversimplify the pain experience, and some patients find it difficult to express their individual, subjective, multidimensional experience of pain as a mark on a line.

There is no simple tool to objectively record the degree and type of pain experienced by a patient. To help address this need, the newly developed PV device was recently introduced for quantitative assessment of pain sensation and perception through direct measurement of pain intensity as a “degree of pain.” In clinical practice, this device has been used to evaluate not only chronic pain, such as lower back pain caused by spondylolisthesis (Lee et al. 2014) or fibromyalgia (Osada et al. 2011), but also to assess acute pain due to the removal of wound dressing materials (Matsumura et al. 2012). They have reported that PV is a useful device to objectively evaluate pain in various fields. The system is based on the supply of alternative painless sensory stimulation equivalent to pain (through the stimulation of Aβ and Aδ sensory nerve fibers) and the measurement of the degree of the stimulation. Because individual pain thresholds are assessed first for accurate subsequent measurement with the method, pain intensity can be quantitatively compared among patients. Therefore, this method enables a more objective assessment in comparison with other generally used methods. However, there are currently no published reports on the use of PV to evaluate VP. Prevention and improvement of oxaliplatin-induced VP is critical to improve patients’ quality of life and support continuation of treatment.

A method to compare two intra-individual coefficients of variations (CVs) considering the paired sampling, different inter-individual variations between measures and some covariates have been proposed (Kiyomi et al. 2016). Two measurements by PV and VAS are performed simultaneously. To evaluate within patient consistency, intra-individual CVs are compared between measures assuming that the pain status of each patient is stable during the study period. The correlated samples and different inter-individual variations due to different scales of the measures should be taken into account in the statistical analysis. This adjustment of covariates improves the estimation of population mean values of the measures. The approach is useful to compare two intra-individual CVs taking into account the paired sampling and different inter-individual variations between measures and some covariates.

To the best of our knowledge, there is no report that has quantified oxaliplatin-induced VP using electrical stimulation. One limitation of this study was using only VAS as a subjective evaluation and not allowing adequate VP assessment. Accordingly, the Neuropathic Pain Questionnaire, Leeds Assessment of Neuropathic Symptoms, and Signs scale and Douleur Neuropathique en 4 Questions should also be compared to confirm the current findings. We think that the drug effects for oxaliplatin-induced VP should be evaluated objectively.

## Conclusions

Although both assessment methods evaluated the same phenomenon, a strong correlation was not observed between the results of each assessment method, and only a weak correlation was observed between PV and VAS. These results suggest that because PV and VAS each measure different factors, both may be needed to adequately evaluate oxaliplatin-induced VP with the aim of aiding treatment. These findings are expected to help with the improvement of VP through the usage of an objective assessment method and to support future clinical trials associated with VP caused by oxaliplatin.
